# Access to palliative care: discrepancy among low-income and high-income countries

**DOI:** 10.7189/jogh.09.020309

**Published:** 2019-12

**Authors:** Arjun Poudel, Bhuvan KC, Shakti Shrestha, Lisa Nissen

**Affiliations:** 1 School of Clinical Sciences, Queensland University of Technology, Queensland, Australia; 2 Sankalpa Foundation Pvt. Ltd., Pokhara, Nepal; 3 School of Pharmacy, University of Queensland, Queensland, Australia; 4 School of Pharmacy, Monash University, Malaysia

Recent advances in technology have led to the expansion of treatment options that can sustain life in circumstances where this was previously impossible [1]. The advancement in treatment options, while a societal success, presents many challenge to health care systems. The greatest challenge of this century is how we care our loved ones who are granted greater longevity but a cure is out of reach. As the global population is ageing and growing, the incidence of chronic disease and life limiting illnesses are also increasing. Patients with life limiting illnesses such as advanced cancer, end stage organ failure, neurodegenerative disease, and AIDS should have access to appropriate health care and basic medications intended for their pain and symptom control, enabling them to live as well as possible, and die with dignity and comfort [2].

The concept of hospice and palliative care emerged in the nineteenth century when western countries went through major demographic and social changes [3]. Hospice care is end-of-life care (often for patients with life expectancy less than 6 months) provided by health professionals and volunteers to provide medical, psychological and spiritual support. The goal of the care is to help people who are dying have peace, comfort and dignity [4]. Palliative care is defined as an approach that improves the quality of life of patients and their families facing the problems associated with life-threatening illness, through the prevention and relief of suffering [5]. Palliative care involves a multidisciplinary approach that includes mental health services, hospice care and pain relief. The initial focus of palliative care was on those dying from cancer; however, the approach of palliative care further contributed to the improvement in the quality of life of people dying with other non-communicable conditions and life threatening infectious diseases [6]. Most high-income countries (HICs) have effective palliative care interventions to respond serious health related sufferings. However, there is little to no access to pain relief or palliative care in low- and middle-income countries (LMICs) [7].

**Figure:**
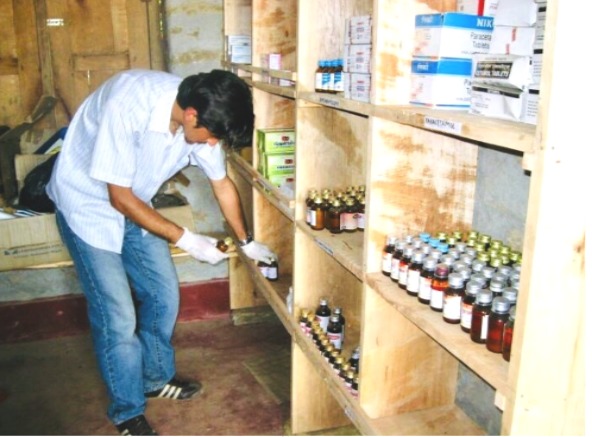
Photo: Accessing the stock of essential medicines in the remote health post (from the collection of Dr Poudel, used with permission).

## PROBLEM STATEMENT

The report from the International Covenant on Economic, Social and Cultural Rights Article 12.1 (1966) states that human right to health is “right of everyone to the enjoyment of the highest attainable standard of physical and mental health”[8]. While “*palliative care”*, the term as such has not been explicitly stated in the statement, access to palliative care, including access to pain relief is acknowledged as a human right [9,10]. Though palliative care is a human right, there are disparities among LMICs and HICs when it comes to accessing and utilisation of palliative care services [11]. The World Health Organisation (WHO) estimates that there were approximately 56.4 million deaths in 2015 of which 70% death were due to non-communicable diseases (NCD) [12]. The prevalence of NCD is high among LMICs. Over three quarters (approximately 30.7 million) of NCD deaths in 2015 occurred in LMICs. Likewise, an estimated 40 million people worldwide need palliative care annually, of which nearly 80% live in LMICs [13]. Unfortunately, only about 14% of such population have access to palliative care services at the end of life which are mostly limited to HICs [13]. A study to map global levels of palliative care development in 234 countries showed that only 20 countries (8.5%) had advanced integrated palliative care provision while most either lacked hospice-palliative care activity (32.1%) or had isolated service (31.6%), highlighting the poor accessibility to palliative care service globally [14]. The United Nations report on progress towards the Sustainable Development Goals (SDG)-3 aim to ensure healthy lives and well-being for all [15]. Improving access to palliative care, including access to pain relief medication such as morphine is fundamental to achieve the SDGs. Morphine has lately been enlisted in the WHO Model List of Essential Medicines but their accessibility and availability are limited in LMICs.

## CHALLENGES AND OPPORTUNITIES

Access to palliative care in LMICs has been hindered by several factors such as the unavailability of pain medications (such as morphine and other opioids) and other treatments, expensive palliative care set up in hospitals, lack of proper clinical system (guidelines) for palliative service, palliative care not receiving enough priority, and a socio-cultural belief system that does not envisage palliative care [16,17]. The absence or limitation of palliative care services in a wider part of the world is also augmented by the disparity in the distribution of narcotic analgesic among countries of varying income. For instance, only 0.03% of total morphine-equivalent opioids distributed worldwide in a year (average of 2010 to 2013 was 298.5 metric tonnes) was supplied to LMICs where 83% of the total population live [7]. Ironically, 94% of the world’s opioids are consumed in HICs [18]. For example, Haiti receives just 1% of its need of pain relievers while the United States imports 31 times the amount of pain relievers it needs [19]. Therefore, opioids and other pain relievers are not available where they are most needed but are abundant where they might be abused. This, however, is not just the only reason for uneven distribution. The demand and supply process is also hindered by the challenges of opiophobia [17] and regulations prohibiting opioid prescribing in many developing nations [20]. Palliative care with oral morphine, a potent pain reliever which is enlisted as an essential medicine by the WHO [21], is not available in most of the LMICs, either because of fears concerning addictive drugs held by authorities, or a failure to acknowledge the needs of the less privileged. The contrast between rich and poor countries seems much larger in ‘pain control’ than in any other facet of medical practice. Hence, individuals in LMICs who need palliative care at the end of life often die with pain and discomfort.

Setting up a palliative care service in LMICs has several challenges which include lack of funding and health care system lacking facility, technologies and human resource required to run palliative care services [22]. Palliative care is integrated into mainstream health care provision in HICs. Funding for palliative care services varies depending on the type of service and the setting in which it is provided [23]. Most of the time they are publicly funded and hence the inequality in access to palliative care is minimised in HICs. With only a few exceptions, palliative care is not incorporated into the health care systems in LMICs. Therefore, there is no allocation of public funds or institutional resources and no reimbursement for services rendered through health insurance programs [24]. Lack of publicly funded palliative care services leads to bearing of such cost by families which causes catastrophic spending in health care, potentially catalysing poverty [11]. Therefore, the issue of palliative care needs to be addressed together with poverty reduction so that it can get global support and garner adequate funding. It highlights the need to build collaboration between institutions of LMICs and that of the developed world [11]. Specialized centers in the HICs that have both expertise and resources in providing palliative care can help train health care providers in LMICs to develop a protocol for palliative care services. Likewise, a multilateral and public-private partnership can be developed to help low-income countries (LICs) produce required pharmaceuticals and other supplies essential for palliative care. Such strategies showed promising results with HIV/AIDS and tuberculosis medications and thus, can be replicated to palliative care as well [25,26].

Similarly, community based services with home-based care (HBC) models have been successful in low-resource settings. Many HBC models were initiated in LMICs such as India, Malawi, Tanzania, Kyrgyzstan, and Myanmar by a small group of people, gradually adding elements of care. The exact reason for the success of HBC remains to be explored. However, these models are known to involve families and volunteers for essential symptom management measures by training them together with supplementation of health care professional for necessary medicines and backup support [27]. In this way, probably the emotional aspects of care for patient tend to fulfil and the direct cost of implementing more staff for care reduces. Another promising approach is to set up a community based palliative care day clinic with support from HICs. HBC models, however, are criticized for the catastrophic health care expenditure in LICs that might push families into poverty cycle and hinder local economic growth [11].

## Future directions

Palliative care services are not integrated within the national health system as a result of which these facilities are either non-existent or have very little coverage in LMICs [28]. This then raises the issues around human rights to health and privilege to die without pain related sufferings. Just because the pain control medications do not make individual live any longer or make them more productive, should not their *human right of not suffering any more pain* be restored? Simply because they are poor, should not they have a privilege to ease their pain at the end of life? The problem in LMICs is mostly the failure of their government to prioritise the need for palliative care at the end of life rather than the issues with affordability. The Lancet Commission comprising an expert panel of palliative care providers propose an essential package (drugs, equipment and training) for LICs that would cost about US$2.16 per capita per year [7]. The package is an achievable goal (amidst other approaches) for a determined government if they prioritise and incorporate pain relief and palliative care in their health care and social development agendas. A socio-cultural belief system about palliative care is another important dimension that needs proper attention. Each society has its own traditional belief system and socio-cultural practices when it comes to treating persons with life-threatening diseases or providing end-of-life care [29]. It is important to address these socio-cultural stakeholders’ belief systems as it influences both the legislators and health care practitioners as well as the individual and the family who demands and utilise palliative care. Additional research and interventions are needed to know more about the socio-cultural belief systems so that country specific palliative care needs are identified and adopted.

## CONCLUSION

Access to palliative care, including access to pain relief, is a human right. Yet, millions of people in LMICs have limited access to this type of care mostly because palliative care services are not integrated within the national health system. Comprehensive palliative care models needs to be implemented in LMICs so that patients with life limiting illnesses can improve their quality of life and die with dignity and comfort.
